# A Population-Based Analysis of Three Treatment Modalities for Malignant Obstruction of the Proximal Colon: Acute Resection Versus Stent or Stoma as a Bridge to Surgery

**DOI:** 10.1245/s10434-016-5247-7

**Published:** 2016-05-24

**Authors:** F. J. Amelung, E. C. J. Consten, P. D. Siersema, P. J. Tanis

**Affiliations:** 1 Department of Surgery, Meander Medical Centre, Amersfoort, The Netherlands; 2 Department of Gastroenterology and Hepatology, Academic Medical Centre, Utrecht, The Netherlands; 3 Department of Surgery, Academic Medical Centre, University of Amsterdam, Amsterdam, The Netherlands

## Abstract

**Background:**

Malignant obstruction of the proximal colon (MOPC) traditionally has been treated with acute resection. However, morbidity and mortality rates following these emergency surgeries are high. Initial bowel decompression by stent placement or stoma construction has been used for distal obstructions as an alternative approach. This study evaluated whether these alternative treatment strategies could be beneficial for patients with a MOPC as well.

**Methods:**

All patients undergoing a colonic resection for a MOPC between January 2009 and December 2013 and who were registered in the Dutch Surgical Colorectal Audit were analyzed.

**Results:**

From the 49,013 patients registered in the DSCA, 1860 (3.8 %) were selected for further analysis. Acute resection was performed in 1774 patients (95.4 %), 44 patients (2.4 %) were treated with initial decompression using stent placement and resection, and 42 patients (2.3 %) with stoma construction followed by resection. Thirty-day mortality was 8.8, 2.4, and 2.4 %, respectively. Mortality was significantly lower after a bridging strategy (stent or stoma) compared with acute resection (*p* = 0.04). Complications following the resection occurred in 39.6% in the acute resection group and in 27.3 and 31.7% in the stent and stoma group, respectively (*p* = 0.167).

**Conclusions:**

Acute resection was performed in the vast majority of patients with obstructive proximal colon cancer and resulted in a 40 % morbidity and 9 % mortality rate. A bridging strategy may be a valid alternative in some of these patients, because a significantly lower postoperative mortality rate was seen in a subgroup of patients initially treated with a stent or stoma.

Colon carcinoma is one of the most frequently encountered malignancies in the western world, and each year its incidence increases.^1^ Up to 9–13 % of all patients with colon cancer present with acute bowel obstruction.^2^–^4^ Approximately 33–54 % of these obstructing tumors are located in the proximal colon.^5^–^7^

Malignant obstruction of the proximal colon (MOPC) is considered a life-threatening condition that requires emergency surgery. Traditionally, MOPC has been treated with acute resection and primary anastomosis, which was deemed safe after several prospective studies showed no difference in mortality or morbidity rates when emergency and elective resections were compared.^8^ Recent studies, however, have shown significantly higher mortality rates in up to 34 % of patients after emergency resections.^9^–^11^

Patients presenting with MOPC often are elderly and in poor physical condition due to several days of reduced intake, vomiting, and weight loss before presentation. These factors are associated with an increased operative risk leading to high mortality rates.^9^,^11^,^12^ Initial colonic decompression using a minimally invasive procedure as a bridge to surgery (BTS) might be an attractive alternative to acute resection. This approach creates time to optimize the patients’ condition and perform oncologic staging, which could prevent unnecessary surgery in palliative patients. A BTS approach can be achieved by endoscopic stent placement at the site of obstruction or by stoma construction proximal to the obstruction.

The BTS approach has been extensively researched for left-sided colonic obstructions. In the recently published European Guideline on colonic stenting, BTS by stent placement in the curative setting is recommended for all patients ≥70 years or with an ASA-score ≥3.^13^ However, <5 % of all literature on colonic obstruction involves stenting in the proximal colon and, to our knowledge, no literature is available on stoma as BTS for MOPC at all.^14^ A recent systematic review comparing stent and acute resection for MOPC suggested lower mortality and morbidity rates when stent placement is used as BTS, but the included studies were small and of low quality.^15^

This study was designed to determine the use and corresponding outcomes of a BTS approach using stent placement or stoma creation in the Netherlands from 2009–2013, based on prospectively registered data. In addition, the outcomes of both BTS strategies were compared to the outcomes following emergency resection.

## Patients and Methods

### Study Design and Population

Data of all patients who underwent a resection for MOPC between 2009-2013 were collected from a prospective national colorectal cancer registry: the dutch surgical colorectal audit (DSCA). This registry includes data for all patients undergoing resection of colorectal cancer in the Netherlands. All Dutch hospitals are obliged to deliver these data, and validity is achieved by control tools in the web-based data entry program, by sending feedback on missing or improbable data, and by annual comparison with the National Cancer Registry on completeness and accuracy.^16^ The database was obtained after approval of the study protocol by the DSCA review board.

Patients were included for analysis when they met the following criteria: (1) symptomatic colonic obstruction, (2) proximal location of the obstruction (cecum, ascending colon, hepatic flexure or transverse colon), and (3) the obstruction was caused by histologically proven colon cancer. After patient selection from the DSCA database, patients were further subdivided into three groups depending on the initial treatment strategy applied: stent placement, stoma construction, or acute resection. When initial decompression using stent placement had failed and emergency surgery was performed, the patient was still analyzed as having undergone stent placement. Patients presenting with perforation and fecal peritonitis were excluded from analyses.

### Data Extraction

The following data were extracted from the DSCA database: patient characteristics (age, gender, ASA score), surgical characteristics (urgency of surgery, resection type, open/laparoscopic approach, type of BTS approach used), data on the primary tumor (pathological TNM-stage, location), overall complication rate, and mortality. Mortality was defined as death within 30 days or during hospital stay after resection. Overall complications were defined as surgical and nonsurgical complications occurring within 30 days or in-hospital. No long-term data or data on complications associated with an initial decompression are registered in the DSCA. Furthermore, data on decision-making regarding treatment approaches is not available, although we know that mainly one regional teaching hospital performed stent as BTS during the study period. Because all data in the DSCA database are anonymous, retrieval of missing data was not possible.

### Outcome Parameters

Patients treated with stent placement, stoma construction, or acute resection were compared for baseline characteristics and outcome parameters. The primary outcome measure was mortality. Other outcome parameters were overall complication rate and the percentage of radical resections.

### Statistical Analysis

Statistical analysis was performed using SPSS statistics 22. Continues variables were described as mean with standard deviation and range. Categorical variables were described as counts and percentages. Fisher’s exact test or the *χ*^2^ test was used for data analysis with categorical variables and one-way ANOVA for analysis of continues variables. Reported *p* values are two-sided and were considered significant when <0.05.

## Results

### Patient’s Characteristics

**Fig. 1:**
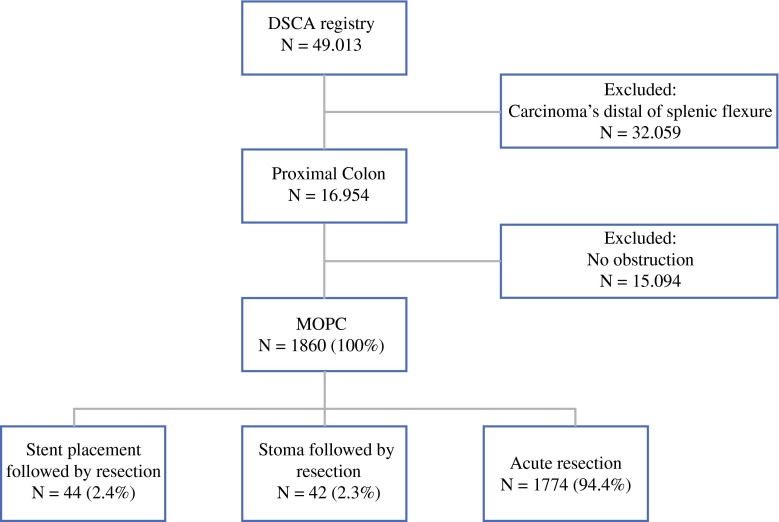
Patient selection from the DSCA database from 2009–2013. *DCSA* dutch surgical colorectal audit, *MOPC* malignant obstruction of the proximal colon

Between January 2009 and December 2013, a total of 49,014 patients were included in the DSCA database; 1860 patients had MOPC and were eligible for the present analysis. Overall, 1774 (95.4 %) patients were treated with acute resection, 44 (2.4 %) patients received a stent and subsequent resection, and 42 (2.3 %) patients had a stoma created as a BTS (Fig. 1). A decrease in the frequency of stent placement was observed from approximately 3.5 % in 2009–2012 to 0.5 % in 2012–2013.

ASA score and gender were similar between treatment groups (Table 1). The stoma group had a significantly lower age (64.9 years) compared with the stent and acute resection groups (69.9 and 71.4 years, respectively, *p* = 0.001). In addition, more tumors were localized in the transverse colon and fewer in the ascending colon in patients treated with stent compared with stoma or acute resection (*p* < 0.001). Significantly more patients had a pT4 stage in the stoma group, whereas M1 stage was similar among treatment groups.

**Table 1:** Baseline characteristics

*N* = 1860	Stent followed by resectio*n* (%) *N* = 44	Stoma followed by resectio*n* (%) *N* = 42	Acute resection (%) *N* = 1774	*p* value Stent versus stoma versus acute resection	*p* value stent/stoma versus acute resection	*p* value stent versus acute resection	*p* value stoma versus acute resection
Male:female	20:24	24:18	858:916	0.49	0.66	0.63	0.29
Mean age (range)	69.9 (53–86)	64.9 (35–89)	71.4 (15–100)	<0.01	0.01	0.43	<0.01
ASA score (*N* = 1830)							
I	8/44 (18.2)	7/42 (16.7)	253/1744 (14.5)	0.74	0.44	0.26	0.51
II	23/44 (52.3)	29/42 (69.0)	877/1744 (50.3)	0.06	0.08	0.52	0.05
III	13/44 (29.5)	6/42 (14.3)	538/1744 (30.8)	0.07	0.09	0.38	0.03
IV	0/44 (0.0)	0/42 (0.0)	75/1774 (4.3)	0.15	0.05	0.41	0.26
V	0/44 (0.0)	0/42 (0.0)	1/1744 (0.1)	0.98	0.82	0.88	0.87
Year^a^ (*N* = 1853)							
2009	9/264 (3.4)	2/264 (0.7)	253/264 (95.8)				
2010	12/330 (3.6)	11/330 (3.3)	307/330 (93.0)				
2011	11/401 (2.7)	11/401 (2.7)	379/401 (94.5)				
2012	9/434 (2.0)	7/434 (1.6)	427/434 (96.9)				
2013	2/415 (0.5)^b^	11/415 (2.7)^b^	402/415 (96.9)^b^				
Tumor localization (*N* = 1860)							
Cecum	6/44 (13.7)	16/42 (38.1)	621/1774 (35.0)	<0.01	0.03	<0.01	0.78
Ascending colon	14/44 (31.8)	6/42 (14.3)	499/1774 (28.1)	0.12	0.35	0.27	0.04
Hepatic flexure	7/44 (15.9)	6/42 (14.3)	268/1774 (15.1)	0.98	1.00	0.61	0.79
Transverse colon 5	17/44 (38.6)	14/42 (33.3)	386/1774 (21.8)	0.07	<0.01	0.19	0.05
pT4 stage (*N* = 1845)	8/44 (18.2)	20/42 (47.6)	589/1759 (33.5)	0.02	0.88	0.02	0.05
pN1–2 (*N* = 1832)	21/44 (47.7)	27/42 (65.9)	1131/1747 (64.7)	0.07	0.13	0.04	0.75
M1 stage (*N* = 1769)	13/43 (30.2)	15/41 (36.6)	478/1712 (27.9)	0.83	0.55	0.52	0.59
Postoperative chemotherapy yes/no (*N* = 1828)				0.46	0.45	0.92	0.21
Adjuvant	12/43 (27.9)	16/42 (38.1)	505/1743 (29.0)	0.43	0.43	0.99	0.16
Palliative	6/43 (14.0)	6/42 (14.3)	242/1743 (13.9)	0.99	0.95	0.90	0.96

^a^Percentage of row total instead of column total

^b^Number of stents placed decreased significantly from 2009–2010–2011 versus 2012–2013, *p* < 0.01. The number of stomas and acute resections increased, *p* = 0.01 and *p* = 0.03, respectively

### Surgical Characteristics

After stent placement or stoma construction, resection was performed in an elective setting in 79.5 and 90.5% of the patients, respectively (Table 2). An emergency resection (<12 h) was performed in four patients of the stent group and in none of the stoma group. Reasons for emergency surgery, despite (an attempt to) colonic decompression, are not registered in the DSCA. However, two patients in the stent group had a registered perforation with fecal peritonitis as a possible explanation of the four emergency resections. Urgent resection (not according to elective planning) was performed in five and four patients of the stent and stoma groups, respectively.

**Table 2:** Surgical characteristics

N = 1860	Stent followed by resectio*n* (%) *N* = 44	Stoma followed by resectio*n* (%) *N* = 42	Acute resection (%) *N* = 1774	*p* value Stent versus stoma versus acute resection	*p* value stent/stoma versus acute resection	*p* value stent versus Acute resection	*p* value stoma versus acute resection
Urgency of surgery (*N* = 1860)							
Elective	35/44 (79.5)	38/42 (90.5)	–	0.16^a^	–	–	–
Urgent (not electively planned)	5/44 (11.4)	4/42 (9.5)	936/1860 (52.8)	0.67^a^	–	–	–
Emergency (<12 h)	4/44 (9.1)	0/42 (0.0)	838/1860 (47.2)	0.04^a^	–	–	–
Interval stent/stoma—resection (d)					–	–	–
Mean (SD)	28.1 (30.9)	109.9 (100.2)	–	0.01	–	–	–
95% CI of mean	8.4–47.7	65.4–154.3	–	–	–	–	–
Range	0–118	0–358	–	–	–	–	–
Surgical procedure (*N* = 1858)							
Ileocecal resection	0/44 (0.0)	2/42 (1.8)	77/1772 (4.3)	0.36	0.37	0.18	0.95
(Extended) right hemicolectomy	30/44 (68.2)	24/42 (57.1)	1423/1772 (80.3)	<0.01	<0.01	0.38	<0.01
Transversectomy	6/44 (13.6)	3/42 (7.1)	107/1772 (6.0)	0.12	0.10	0.30	0.42
(Extended) left hemicolectomy	8/44 (18.2)	7/42 (16.7)	87/1772 (4.9)	<0.01	<0.01	<0.01	<0.01
(Sub)total colectomy	0/44 (0.0)	4/42 (9.5)	32/1772 (1.8)	0.01	0.06	0.39	<0.01
Other	0/44 (0.0)	2/42 (4.8)	46/1772 (2.6)	0.29	0.85	0.39	0.25
Surgical approach (*N* = 1858)				0.03	0.04	<0.01	0.98
Open	34/44 (77.3)	38/42 (90.5)	1621/1772 (91.5)				
Laparoscopic	10/44 (22.7)	4/42 (9.5)	150/1772 (8.5)				
Anastomosis constructed (*N* = 1845)	42/44 (95.5)	38/42 (90.5)	1506/1759 (85.6)	0.12	0.05	0.10	0.32
Stoma constructed after resection (*n* = 1600)							
No	38/43 (88.4)	34/42 (81.0)	1454/1751 (83.0)	0.61^b^	0.68^b^	0.14^b^	0.56^b^
Diverting ileostomy	2/43 (4.7)	4/42 (7.1)	69/1751 (3.9)	0.56	0.37	0.67	0.09
End ileostomy	1/43 (2.3)	0/42 (0.0)	166/1751 (9.5)	0.03	<0.01	0.15	0.03
Diverting colostomy	1/43 (2.3)	3/42 (7.1)	13/1751 (0.7)	<0.01	<0.01	0.19	<0.01
End colostomy	1/43 (2.3)	2/42 (4.8)	47/1751 (2.7)	0.71	0.64	0.30	0.47

^a^ *p* value is for stent versus stoma, because emergency resection is never elective

^b^ *p* value is for stoma construction yes/no in the different groups

The interval between the initial colonic decompression and eventual (elective) resection differed significantly between stent placement and stoma creation (28.1 vs. 109.9 days, *p* = 0.01). More transversectomies and left hemicolectomies were performed in the BTS groups compared with the acute resection group, which was related to differences in tumor localization. Furthermore, a laparoscopic approach was significantly more frequently used after stent placement when compared to stoma construction and acute resection (22.7 vs. 9.5 vs. 8.5 %, respectively, *p* = 0.027). No differences were found in the total number of primary constructed anastomoses, number of protective stomas after resection, or the use of postoperative chemotherapy.

### Outcome Parameters

Mortality in the stent, stoma, and acute resection groups was 2.4, 2.4, and 8.8 %, respectively. When stent and stoma patients were analyzed together as BTS group and compared with acute resection, the difference in mortality was statistically significant (*p* = 0.04). Mortality rates for different subgroups based on age, ASA score, and type of resection for patients who underwent acute resection are shown in Table 3. Subgroup analyses could not be performed for the stent and stoma groups due to the low number of events.

**Table 3:** Surgical outcomes

*N* = 1860	Stent followed by resection *n* (%) *N* = 44	Stoma followed by resectio*n* (%) *N* = 42	Acute resection (%) *N* = 1774	*p* value stent versus stoma versus acute resection	*p* value stent/stoma versus acute resection	*p* value stent versus acute resection	*p* value stoma versus acute resection
Complications within 30 days (*N* = 1850)	12/44 (27.3)	13/41 (31.7)	699/1765 (39.6)	0.17	0.07	0.05	0.22
Reintervention (*N* = 1082)	8/28 (28.6)	10/29 (34.5)	240/1025 (23.4)	0.32	0.20	0.63	0.18
Reintervention indication				0.92	0.89	0.95	0.57
Anastomotic leakage	2 (22.2)	5 (50.0)	111 (42.5)	0.42	0.63	0.32	0.63
Abscess	1 (11.1)	1 (10.0)	29 (11.1)	0.99	0.94	0.90	0.92
Re-bleeding	1 (11.1)	0 (0.0)	16 (6.1)	0.59	0.88	0.46	0.42
Ileus	1 (11.1)	0 (0.0)	15 (15.7)	0.58	0.93	0.42	0.44
Fascia dehiscence	2 (22.2)	4 (40.0)	41 (15.7)	0.12	0.07	0.47	0.04
Other	0 (0.0)	0 (0.0)	2 (0.8)	0.30	0.37	0.64	0.13
Type of reintervention (*N* = 262)							
Radiological	1 (12.5)	1 (10.0)	26 (6.2)	0.91	0.67	0.55	0.82
Laparoscopy	1 (12.5)	1 (10.0)	2 (1.6)	<0.01	<0.01	<0.01	<0.01
Laparotomy	4 (50.0)	5 (50.0)	196 (81.6)	0.21	0.08	0.09	0.24
Other/unknown	2 (25.0)	3 (30.0)	16 (6.6)	0.08	0.03	0.05	0.12
Completeness of resection (*N* = 1738)							
R0	37/40 (92.5)	33/39 (84.6)	1524/1659 (91.9)	0.26	0.30	0.97	0.13
R1	1/40 (2.5)	6/39 (15.4)	76/1659 (4.6)	<0.01	0.08	0.60	0.01
R2	2/40 (5.0)	0/39 (0.0)	58/1659 (3.5)	0.43	0.65	0.50	0.22
30-day mortality (*N* = 1843)	1/42 (2.4)	1/42 (2.4)	155/1759 (8.8)	0.17	0.04	0.16	0.17
≤70 years	–	–	36/815 (4.4)				
>70 years	–	–	118/947 (12.5)^a^				
ASA 1–2	–	–	59/1186 (5.0)				
ASA 3–5	–	–	97/629 (15.4)^b^				
Ileocecal resection/right hemicolectomy	–	–	125/1489 (8.4)				
Transversectomy/left hemicolectomy/subtotal colectomy	–	–	26/240 (10.8)^c^				

^a^ *p* value <70 versus 70 years: <0.01

^b^ *p* value ASA 1–2 versus ASA 3–5: <0.01

^c^ *p* value right-sided colonic resection versus left-sided colonic resection: 0.07

The number of complications within 30 days after resection was equal between treatment groups. However, if stent placement was compared separately to acute resection, a borderline significant difference in favor of stent was found (27.3 vs. 39.6 %, *p* = 0.051) (Table 3). Reintervention rate was not significantly different between the treatment groups; in addition, the type of complication requiring reintervention did not differ. Postoperative complication rate for both the stent and stoma groups were lower when initial decompression was clinically successful and followed by elective resection. When acute resection was compared with only those patients in whom decompression with stent or stoma before resection was successful, complication rates did significantly differ (39.7 % after acute resection, 27.0 % after stoma and elective resection, and 22.9 % for stent and elective resection, *p* = 0.043).

No significant difference was found between treatment groups with regard to completeness of resection; however, more microscopic irradical resections (R1) were seen in the stoma group (15.4 %) compared with the stent (2.5 %) and acute resection (4.6 %) groups.

## Discussion

This large, population-based analysis of MOPC demonstrated that acute resection was performed in 95 % of the patients, with a primary anastomosis rate of 86 %. A decompressing intervention as BTS was performed in only 5 % of the patients. Mortality was significantly lower after a bridging strategy compared with acute resection. In addition, mortality rates after acute resection were approximately three times higher in patients ≥70 years or with an ASA-score ≥3.

These observations are clinically important, because they may lead to a more patient-tailored treatment strategy. Our findings suggest that mortality rates could have been lower if more patients had been treated with a BTS, especially elderly patients with one or more comorbidities. Interestingly, a decrease in stent placement for MOPC from 3.5 % in 2009 to 0.5 % in 2013 was seen. This change in decision-making is probably the result of the premature closure of two Dutch randomized, controlled trials comparing stent with emergency surgery. Both trials were prematurely closed due to the high incidence of stent-related complications, making physicians more hesitant towards stent placement.^17^,^18^ Unfortunately, definitive conclusions cannot be drawn solely based on the outcomes of the present study, because this is an observational cohort study with a risk of selection bias. It is not known from the DSCA database how patients were selected for the different treatment strategies. Ninety-two hospitals included patients, and treatment approaches highly depended on local expertise. Despite these methodological issues, the present data represent the best available evidence, because no randomized trials have been performed and the present study is the largest comparative series available.

The current findings agree with observations for left-sided colonic obstructions in the literature.^19^ It may be that treatment strategies for MOPC and left-sided malignant colonic obstruction should be identical. Nonetheless, the recently published European guideline on colonic stenting recommends a bridging strategy only for left-sided obstructing colon cancer patients with an increased operative risk (age ≥70 years/ASA-score ≥3).^13^ The present data support such an approach for MOPC as well, which could ultimately lower mortality rates, especially in elderly and frail patients.

Several studies have tried to identify independent predictors of mortality when performing emergency surgery for large-bowel obstruction. Tekkis et al. analyzed a group of right- and left-sided colonic obstructions and found significantly higher mortality for patients ≥70 years or with an ASA-score ≥3.^20^ Kobayashi et al. evaluated a cohort of 15,275 patients who underwent a right hemicolectomy and found an odds ratio of 2.32 in patients with ASA scores ≥3.^9^ In addition, several other studies have associated ASA score ≥3 and advanced age with higher mortality after colorectal surgery; however, no studies have specifically identified risk factors for MOPC.^11^,^12^,^21^

Studies on MOPC are scarce, and to our knowledge, no other articles on decompressing stoma as BTS for MOPC have been published. A recent systematic review comparing stent as BTS with acute resection in MOPC patients showed a mortality rate of 0% in the stent group versus 10.8% in the acute resection group (*p* = 0.009). In addition, stenting was associated with lower morbidity rates and fewer permanent stomas. These retrospective results are similar and supportive to those found is our prospective analysis.^15^ However, these data should be interpreted with caution, because all included studies in the systematic review were cohort studies. Kye et al.^22^ recently published the first study directly comparing stent and acute resection as treatment options for MOPC. Similar to our outcomes, they found laparoscopic resection rate to be significantly higher following stent placement. In addition, significantly more lymph nodes were harvested in the stent group. Contrary to our study, however, they did not find a significant difference in 30-day mortality and morbidity.^22^ A possible explanation for this discrepancy could be that they included considerable fewer patients than the current study and might have lacked power to demonstrate a significant difference.

Based on the currently available data, a bridging strategy might be the preferred initial approach in elderly and/or frail patients, where the risk of emergency surgery might be relatively high. However, stent placement for left-sided obstructing colon cancer has been used with reserve during the past years, which is probably due to fear about stent-related complications and uncertainty about the oncologic long-term outcomes.^23^^,^^24^ Initially, based on retrospective data, stent placement for left-sided obstructing colon cancer was thought to be a promising alternative to acute resection. However, several prospective trials had to be closed due to stent-related complications.^17^,^25^ The high technical and clinical success rates in retrospective studies might have been due to selection bias, where only patients with a subtotal, instead of a total occlusion were treated with stent placement. It is important to realize that this also could be the case for MOPC, because no prospective data are available yet. In addition, the long distance from the anus and the tortuosity of the bowel make proximal stenting considerably more difficult than in the distal colon.^14^,^26^,^27^ In line with this, higher technical failure rates have been reported for proximal stenting, which is most commonly caused by an inability to pass the guidewire through an angulated colon, such as the hepatic flexure.^27^

The DSCA database has a high participation rate from Dutch hospitals (>95%) and presents a good reflection of general practice in surgery for colorectal cancer in the Netherlands.^1^ A few limitations should be kept in mind. DSCA data are registered anonymously, making it impossible to retrieve missing values from the original patient files. Furthermore, because only patients with a colorectal resection are included in the DSCA database, patients in whom a stent was placed but never underwent colonic resection were excluded. Another limitation is that complications and hospital stay related to initial decompression are not available in the registry; this might have had a positive influence on the overall outcomes in the BTS treatment groups.

Keeping these considerations in mind, this study indicates a possible advantage for stent or stoma as a BTS in patients with MOPC compared with acute resection, and high-risk patients potentially benefit most from such a strategy. Our data suggest that the current recommendation for stenting in left-sided colon cancer (ESGE guideline^13^) can be extended to proximal obstructions. A decompressing stoma can be considered an alternative in patients with high operative risk if stenting is not technically feasible or in locally advanced tumors. To optimize a patient-tailored treatment strategy, future research should be focused on identifying more predictors to enable better selection of subgroups of patients who benefit most from a specific treatment strategy.
